# A Systematic Review on Gluten-Free Bread Formulations Using Specific Volume as a Quality Indicator

**DOI:** 10.3390/foods10030614

**Published:** 2021-03-13

**Authors:** Jordanna S. Monteiro, Priscila Farage, Renata Puppin Zandonadi, Raquel B. A. Botelho, Livia de L. de Oliveira, António Raposo, Faiyaz Shakeel, Sultan Alshehri, Wael A. Mahdi, Wilma M. C. Araújo

**Affiliations:** 1 Department of Nutrition, Faculty of Health Sciences, University of Brasilia, Brasilia 70910-900, Brazil; jordanna.santosmonteiro@gmail.com (J.S.M.); renatapz@unb.br (R.P.Z.); raquelbabotelho@gmail.com (R.B.A.B.); liviadelacerda@gmail.com (L.d.L.d.O.); 2 Faculty of Nutrition, Federal University of Goias, Goiânia 74690-900, Brazil; priscilafarage@ufg.br; 3 CBIOS (Research Center for Biosciences and Health Technologies), Universidade Lusófona de Humanidades e Tecnologias, Campo Grande 376, 1749-024 Lisboa, Portugal; 4 Department of Pharmaceutics, College of Pharmacy, King Saud University, Riyadh 11451, Saudi Arabia; faiyazs@fastmail.fm (F.S.); salshehri1@ksu.edu.sa (S.A.); wmahdi@ksu.edu.sa (W.A.M.); 5 Department of Pharmaceutical Science, College of Pharmacy, Almaarefa University, Riyadh 11597, Saudi Arabia

**Keywords:** breadmaking, gluten-free bread, gluten-free bread external characteristics, gluten-free bread internal characteristics specific volume

## Abstract

This study aimed to perform a systematic review on gluten-free bread formulations using specific volumes as a quality indicator. In this systematic review, we identified 259 studies that met inclusion criteria. From these studies, 43 met the requirements of having gluten-free bread with a specific volume greater than or equal to 3.5 cm^3^/g. Other parameters such as the texture profile, color (crumb and crust), and sensory analysis examined in these studies were presented. The formulations that best compensated the lack of the gluten-network were based on the combination of rice flour, rice flour with low amylose content, maize flour, rice starch, corn starch, potato starch, starch with proteins and added with transglutaminase (TGase), and hydrocolloids like hydroxypropylmethylcellulose (HPMC). Of the 43 studies, three did not present risk of bias, and the only parameter evaluated in common in the studies was the specific volume. However, it is necessary to jointly analyze other parameters that contribute to the quality, such as texture profile, external and internal characteristics, acceptability, and useful life of the bread, especially since it is a product obtained through raw materials and unconventional ingredients.

## 1. Introduction

Wheat is the only cereal that contains gliadins and glutenins in adequate concentrations to form gluten. The gliadins contribute primarily to the dough’s viscosity, and glutenins are responsible for dough elasticity [1,2,3,4,5,6]. In breadmaking, the mechanical energy supplied during the mixing process favors the hydration of prolamins (gliadins and glutenins) and induces conformational changes of these proteins. Such structural changes lead to covalent (S-S) formation and non-covalent bonds, and hydrogen bonds that form gluten. Gluten can retain the fermentation gases, thus responding by the bread’s volume, texture, and softness. It displays important technological properties explaining its extensive use in the food industry as raw material and food additive [7]. 

Parallel to this, the literature shows that reactions exist against gluten in predisposed individuals [4,8,9,10]. Gluten-related disorders (GRD) include three primary forms of gluten reactions: allergic (wheat allergy), autoimmune disorders (celiac disease, dermatitis herpetiformis, gluten ataxia), and possibly immune-mediated (gluten sensitivity) [4,11]. All of these conditions require following a strict gluten-free diet (GFD) as a treatment. In addition to GRD individuals, their relatives follow the GFD to support GRD individuals’ treatment and avoid food cross-contamination. Also, individuals without GRD have been following GFD believing in its potential health benefits, despite the lack of scientific evidence [12,13,14]. Therefore, about 10% of the worldwide population follow a GFD [15,16,17]. Gluten-free foods (GFF) are those that the gluten level does not exceed 20 ppm in total [18]. There are specific regulations on gluten-free labeling worldwide. Most of them are based on the Codex Alimentarius Standard 118-1979 and recommend following good manufacturing practices to prevent gluten cross-contamination, ranging from country to country. The European Union, United States, and Canada follow Codex’s limits for GFF (20 ppm) [19,20]. In Argentina, the threshold set for GFF is 10 ppm [21]. In Australia and New Zealand, legislation is stricter and states that to be considered “gluten-free”, food must not contain detectable gluten [22,23]. In Brazil, the legislation sets the obligatoriness, including a statement regarding the presence or absence of gluten in the label of industrial products. However, it does not address the tolerable gluten limit [24].

Despite the demand for gluten-free (GF) products and flours (bakery, pastries, cakes, desserts and ice-creams, ready meals, dairy/dairy alternatives, meat/meat alternatives, condiments, seasonings and spreads, pasta and rice) [25], studies have shown that among GF products, bread is the most required product by people that suffer GRD [26,27,28,29]. However, studies showed limitations on gluten-free bread (GFB) from the technological and sensorial aspects such as pleasing appearance, texture, mouthfeel, and low crumb softness of GFB [30,31,32,33,34]. 

The quality of the bread can be determined by physical, chemical, microbiological, sensory analysis, external loaf characteristics (dimensions, specific volume, the color of the crust, shape, and symmetry), and internal loaf characteristics (thickness of the crust, the color of the crumb, size, and the number of alveoli and crumb texture). Reviews were performed on gluten-free bread quality considering nutritional, sensory, and technological aspects, ingredients [31,35,36,37,38,39,40,41,42,43], but only considered the specific volume as one of the aspects to evaluate GFB [43]. The specific volume is one of the most important indicators of bread’s technological quality, strongly influencing consumer choice. It is used to express the technological aptitude of a formulation for bread production [44,45,46,47,48,49]. Considering that the literature does not yet have a publication on the quality of GFB assessed by specific volume, this study aimed to perform a systematic review on gluten-free bread formulations using the specific volume as a quality indicator.

## 2. Materials and Methods

This systematic review was reported according to the Preferred Reporting Items for Systematic Reviews and Meta-Analyses (PRISMA) Checklist [50] and Guidance of the European Food Safety Authority [51].

### 2.1. Eligibility Criteria

#### 2.1.1. Inclusion Criteria

The inclusion criteria were experimental studies that evaluated the technological, physical-chemical, and/or sensory properties of gluten-free bread (GFB) and presented specific volume above 3.5 cm^3^/g as a GFB quality parameter [52,53]. Studies show that commercial wheat bread’s specific volume varies between 3.5 and 5.5 cm^3^/g [48,54,55,56,57,58,59]. Therefore, we used this minimum value for gluten-containing bread as a specific volume (3.5 cm^3^/g) as a cut-point to evaluate GFB since industries and researchers try to achieve GFB formulations similar to their gluten-containing counterpart. There were no language and time restrictions (from inception to 2 January 2021). 

#### 2.1.2. Exclusion Criteria

The following exclusion criteria were applied: (1) reviews, letters, conference summaries, case reports, short communications, and books; (2) studies of other food products; (3) studies that evaluated only oat flour, bran, or beta-glucan; (4) studies without the physical–chemical, sensory, or technological characteristics of the loaves; (5) clinical studies; (6) studies that evaluated only wheat starch; (7) studies with cereals that present prolamins, such as wheat, rye, barley, which are toxic for celiac and GRD patients; (8) studies that evaluated only GFB dough; (9) studies that used microwave cooking, or steam among other non-conventional methods; (10) studies that did not specify the baking bread method; (11) studies that used commercial GF mixtures without describing their ingredients. Studies that evaluated oat products because of the possible risk of gluten contamination were excluded. Although the European Commission stated that “oats contained in a food presented as gluten-free or very low gluten must have been specially produced, prepared and/or processed in a way to avoid contamination by wheat, rye, barley, or their crossbred varieties and the gluten content of such oats cannot exceed 20 mg/kg” [19], worldwide legislation and the literature on oat safety for GRD individuals are not homogeneous and not recommended for GRD individuals [60,61,62,63]. We excluded studies that evaluated unleavened, flatbread, or French bread because their external and internal characteristics are different from the loaves type [1,25,26].

### 2.2. Information Sources

Detailed individual search strategies for each database were developed for Science Direct, Scopus, Springer link, Web of Science, and Wiley Online Library. We conducted partial gray literature research with Google Scholar and ProQuest Dissertations and Theses Global. The last search across all databases was updated on 2 January 2021. The lists of references of the selected articles were manually examined for full-text reading for possible relevant studies that could have been lost during the database’s electronic search. The list of keywords and the appropriate combinations of truncation and words were selected and adapted for each database search. All references were managed by Endnote desktop software X7 and removed duplicate hits (Supplementary Materials—Table S1).

### 2.3. Study Selection

The selection of the studies was conducted in two phases. In phase 1, two reviewers (JSM, PFG) independently reviewed all references’ titles and abstracts identified from databases. Articles that did not meet the eligibility criteria were discarded. In phase 2, the same reviewers (JSM, PFG) applied the eligibility criteria to the selected articles’ full texts. In both phases, two reviewers discussed the issue in cases of disagreement until a consensus. In situations where there was no consensus, a third reviewer (WMCA) made the final decision. The final selection was always based on the complete text of the publication. The JSM examiner critically evaluated the list of references of the selected studies. Two reviewers (JSM, PFG) extracted data. The third examiner (WMCA) recommended additional studies from the lists of references for full-text reading for possible relevant studies that could have been lost during the database’s electronic search.

### 2.4. Data Collection Process

The following characteristics were collected from the selected articles: authors and year of publication, formulations, the loaves’ physical characteristics (specific volume, texture—hardness, springiness, cohesiveness, resilience, chewiness, color—crust, and crumb, crumb structure), sensory analysis, and water (% on a flour basis). We constructed a table to compare gluten-free bread formulations (with specific volume above 3.5 cm^3^/g) using the specific volume as a quality indicator. 

### 2.5. Risk of Bias (RB)

Based on instructions found in the Guidance of European Food Safety Authority [51], a specific instrument was created to evaluate RB for this study using well-established classical and literature criteria and expert guidance. The bias risk of the selected studies (*n* = 43) assessment instrument included nine questions: (1) characterization of the raw material, ingredients, and gluten substitutes; (2) physical characteristics of the bread; (3) sensorial analysis of the product; (4) function of each ingredient; (5) experimental design; (6) statistical test; (7) results answer the main question. The risk of bias was categorized as High when the study reached up to 49% score “yes”, Moderate when the study reached 50% to 69% score “yes”, and Low when the study reached more than 70% score “yes”.

## 3. Results

### 3.1. Study Selection and Characteristics 

**Figure 1:**
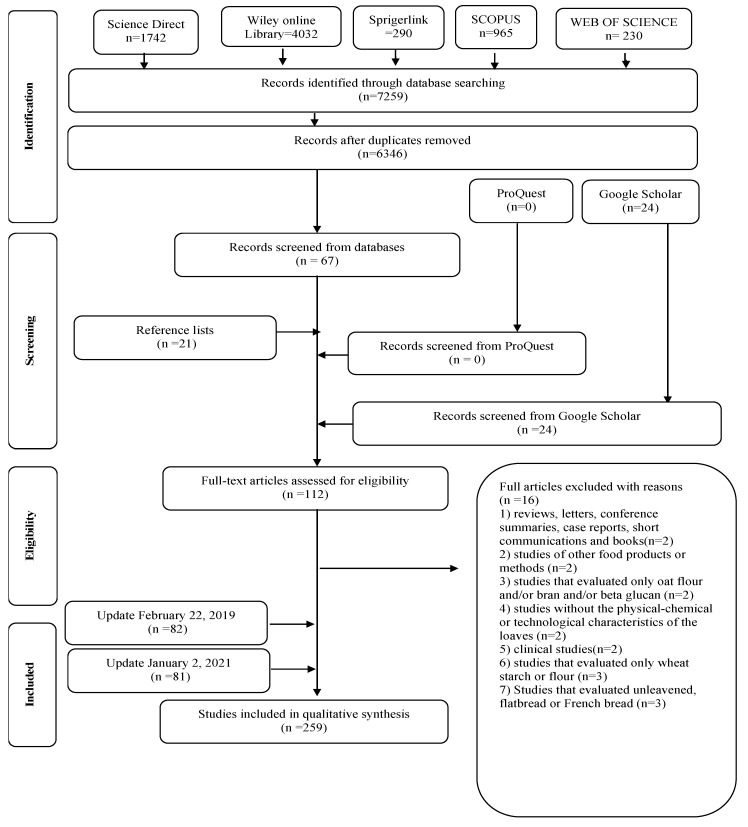
Flow diagram of literature search and selection criteria (Adapted from Preferred Reporting Items for Systematic Reviews and Meta-Analyses—PRISMA).

In all searched electronic databases, 6346 articles were identified in the 1st round performed on 1 June 2016. In Phase 1, 67 articles were selected for their potential interest in Phase 2. Twenty-four articles were identified from the grey literature “Google Scholar (*n* = 24),” and twenty-one were identified in the reference lists (*n* = 21). Specialists suggested reading the other three articles. Thereby, 115 articles were selected for a full reading. Of these, 112 articles were included in the qualitative synthesis. An updated search conducted in February 2019 selected 82 new articles. Another updated search conducted in January 2021 selected 81 new articles. However, 16 of the total were excluded because, after a full read, they did not meet inclusion criteria (Figure 1). Therefore, the total number of studies published from 1976 to 2021 and analyzed based on the review criteria was 259 (Figure 1). The studies were developed in 83 countries. The articles were written in English (*n* = 251; 96%); 4 (1.5%), in Spanish; 3 (1.5%), in Portuguese; 1 (0.3%), in Polish.

### 3.2. Gluten-Free Bread Formulations

Among the 259 evaluated studies, 66% (*n* = 170) used rice flour (*Oryza sativa* L.) as the main ingredient, followed by cornflour (18%; *n* = 46), buckwheat (13.5%; *n* = 35), and soybean (11.5%; *n* = 30). Quinoa and sorghum flours were used approximately in 9% (*n* = 24 and *n* = 23) of the studies. Millet flour was used in 5.4% (*n* = 14) of the studies. Amaranth, chickpea, and tapioca flour in approximately 4% (*n* = 11). Teff flour was used in 3.5% (*n* = 9). The other studies have evaluated the potential of bread making of beans, oats, carob, peas, lupine, corn gluten meal, pine nuts, acorn, babassu, pumpkin, lentil, sweet potato, potato, unripe banana, ripe banana, chia, yacon, nuts, Hemp (*Cannabis sativa* subsp. *sativa*), broken rice berry flour, brown algae, prosopis nigra flour, and defatted hazelnut flour.

Among the 259 analyzed studies, we found that the values for the specific volume of GFB, when determined, ranged from 1.3 to 7.58 cm^3^/g (mean = 4.42 ± 1.06 cm^3^/g). Considering this variation interval, we selected the studies whose results for the specific volume were greater than or equal to 3.5 cm^3^/g, considered a bread quality parameter [52,53]. Therefore, 43 studies (17%) met this criterion (bread with a specific volume greater than or equal to 3.5 cm^3^/g): 3 studies produced bread formulations with a specific volume greater than 7 cm^3^/g; 12 studies produced bread with 5 to 6 cm^3^/g; 13 produced bread with 4 to 5 cm^3^/g; 15 produced bread with 3.5 to 4 cm^3^/g (Table 1).

The formulations that produced bread with a specific volume >7.0 cm^3^/g were based on MS and 80% of water; mixture of rice flour (RF) and maize starch (MS) (50–50%), 5% egg protein substitution (R5), and 90% of water; a mixture of maize flour (MF), MS, and 100% water, all of them added of 2% hydroxypropylmethylcellulose (HPMC). For the crust thickness, the authors indicated the value of 3.99 and for the crumb color the values of L* = 69.79; a* = 3.08 and b* = 19.34 [93]. For the color of the crust, the results obtained were L* = 82.09; a* = 2.64 b* = 19.32 [82]. Gluten-free breads (GFB) with specific volumes equal to 7.58 and 7.14 cm^3^/g showed the following values for the texture profile: hardness (N) 1.44 and 1.25; springiness, 1.011 and 0.95; resilience, 0.493 and 0.41; cohesiveness, 0.754 and 0.56 [66,82].

The formulations that produced GFB with specific volumes from 5 to 6.9 cm^3^/g were composed of different varieties of RF with a change in amylose level, combinations of MS and RF and modified starches, MS and different proteins, native banana starch, protease, and HPMC, flour improver, transglutaminase (TGase), and albumin [27,28,29,30,31,32]. Water absorption varied from 56.5 to 120%. Of these 12 studies, 9 determined the texture profile, but the evaluated parameters were not the same. In general, the values for hardness varied between 0.70 N–22.34; for springiness, 0.95–1.03; 0.45–0.51 for cohesiveness; 0.21–13.61 for resilience; 13.83–583.6 for chewiness (Table 1) [40,46,47,48,67,89,95,98].

Paciulli et al. [89] determined the crust color (L* = 77.5, a* = 2.8; b* = 17.1) and Paciulli et al. [32] and Matos and Rosell [83] determined the crumb color (L* = 76.1/81.50; a* = 1.5/−1.53 (green hue); b* = 11.8/6.47 (yellow hue). The core color showed good lightness. Matos and Rosell [83] also conducted the descriptive sensory analysis with ten trained panelists who assigned the following grades: crumb appearance (3.17), taste (3.33), odor (2.83), on a scale from 0 to 5 points. Roman et al. (2019a) conducted the sensory analysis with 83 volunteers (16 to 65 years), using a scale ranging from “extremely like—9” to “extremely dislike—1”. They obtained the following data: appearance (6.5), odor (6.0), flavor (5.3), texture (5.8), and overall liking (6.0). The other two authors, Nishita et al. [84] and Nishita and Bean [85] reported that the characteristics of the kernels were, respectively, very good and good [85]. In the first study, sensory analysis of the GFB was conducted with 57 tasters who classified it as “slightly disliked” bread (4.2 on a 0–9 scale) (Table 1). 

For the GFB with specific volumes from 4.0 to 5.0 cm^3^/g, formulations were prepared with unripe banana flour, MS, RF, sorghum flour (SF), HPMC, CGTase, different types of starch, fibers, egg whites, among other ingredients. Water absorption varied from 70 to 130%. The crumb hardness values varied from 0.88 to 5.1 N; for springiness, from 0.57 to 8.0; for cohesiveness (0.47–1.0) and for chewiness (0.27–3.7). The acceptability test with 28 panelists indicated a mean acceptance equal to 4.0 ± 2.0 for the formulation proposed by Crockett et al. [102]. For Southgate et al. [97], the sensory acceptability analysis developed with students from the Baking and Pastry Program of the Federal Institute of Education was equal to 6.56 (0 to 9). The texture was considered dry, coarse, sponge-like, sandy, foamy, and the flavor beany. For color and texture of the GFB shell and kernels, variations between 21.77 to 84 were observed for luminosity (L*); for chromaticity, a* varied between −1.53 and 117; and b* between −6.47 and 19.34 (Table 1). 

The formulations proposed by the 15 studies whose GFB had a specific volume greater than 3.5 and less than 4.0 cm^3^/g were based on SF, potato starch (PS), buck-wheat flour (BWF), RF-phosphorylate, MF-Zeína, carob flour, cowpea flour, blend of millet and soybean. The used additives were HPMC, sodium alginate, xanthan gum (XG), TGase, collagen fiber, and different proteins. For these formulations, the texture profile showed the following variation: hardness, ~0.2 a 159N; springiness, 0.230 a 0.98; cohesiveness, 0.057 a 7.3; resilience, 0.049–0.64 [68,72,76,90,91,103]. In four studies, color parameters were determined with a variation of L* = 40.10 to 74; a* = 2.50 to 5.17 and b* = 14.06 to 26.08 for the crust. For the crumb, the obtained values were: L* = 21.77 to 74.21; a* = −1.02 to 117.54; b* 6.75 to 98.87 [69,72,79,103]. Only two studies conducted a sensory analysis of the products, and the acceptability of GFB was between 70 to 75% [72,103].

## 4. Discussion

The first publication objectively compares the results obtained for the specific volume of GFB and some of its external and internal characteristics (crust and crumb) resulting from the formulations proposed in studies published until 2 January 2021. 

To search for the best GFB formulations based on specific volume, we included the studies in which GFB had a specific volume above 3.5 cm^3^/g, as a GFB quality parameter [52,53] (Table 1). Studies show that commercial wheat bread’s specific volume varies between 3.5 and 5.5 cm^3^/g [48,54,55,56,57,58,59]. 

Only 7% (*n* = 3) of the studies proposed formulations whose specific volume of GFB was greater than 7.0 cm^3^/g. In comparison, 58% (*n* = 25) of the studies proposed formulations whose specific GFB volume varied between 4.0 and 6.0 cm^3^/g, while 35% (*n* = 15) of the studies proposed formulations whose specific GFB volume was greater than 3.5 and less than 4.0 cm^3^/g. Only 46% (*n* = 20) determined the parameters for texture profile of the crumb (cohesiveness, elasticity, hardness, etc.); 28% (*n* = 12) determined crust color, crumb color or both; only 16% (*n* = 7%) performed a sensory analysis of the products. 

The specific volume of a loaf is a good indicator of bread quality and is related to the amount of water retained in the network. Accurately represents the volume variation of bread prepared according to different formulations and methods. It represents the ability of gluten strands to retain enough gas released during fermentation and dough proofing. It is an objective characteristic, and when associated with density, it shows the relationship between the solids content and the fraction of air in the baked dough. Dough with a high density and/or with a low specific volume present an unpleasant aspect to the consumer, usually associated with high moisture content, difficulty chewing, and negatively compromised flavor and aroma [47,48,49]. However, sometimes consumers are looking for natural GFB, which is not an important and discriminatory disadvantage, depending on the consumer choice.

Comparing the results obtained by Belorio & Gómez [66], Pico et al. [93] and Martínez and Goméz [25,26] with those of Esteller and Lannes [54,55], who determined the specific volume for samples of different commercial brands of bread loaf, and based on wheat flour, we observed that the specific volume of GFB was higher, possibly indicating the quality of the proposed formulations. Aoki et al. [47], Sahágun et al. [95], Roman et al. [104], Roman et al. [40], Roman et al. [94], Han et al. [75], Berta et al. [67], Bravo-Núñez et al. [48], Hernández-Aguirre et al. [49], Aoki [65], Horstmann et al. [76], Paciulli et al. [89], Matos and Rosell [83], Storck et al. [98], Nishita and Bean [85], and Nishita et al. [84] also proposed gluten-free (GF) formulations that resulted in GFB with a specific volume higher than the average found by Esteller and Lannes [54], which was 4.10 ± 0.19.

The choice of RF is due to its hypoallergenic characteristics, low content (2.5–3.5%) of prolamins, pleasant taste, white color, besides other advantages like economic advantages and being widely available [3,105,106,107,108,109]. The rice proteins are mainly hydrophobic and insoluble. This insolubility does not allow viscoelastic dough formation, necessary for carbonic gas trapping during leavening. Besides, it does not have a profile of prolamins similar to wheat’s prolamins. As a result, bread presents low volume with improper bread crumbs and a high aging rate. On the other hand, zein is a protein found in maize. It shows hydrophobicity as it includes many hydrophobic amino acid residues (leucine, proline, alanine, and phenylalanine) in its amino acid composition [1,37,85,110,111]. 

Nishita and Bean [85] showed that different rice varieties’ physical–chemical properties affect bread’s technological quality. This fact is related to the amylose and amylopectin levels in each type of grain. Short and medium grains usually have a lower level of amylose and lower gelatinization temperature (70–74 °C), being more appropriate for producing GFB. Aoki et al. [47] assessed the breadmaking potential of 19 rice varieties (19 rice flour samples), containing amylose contents ranging from 9.6 to 22.3%. They identified that the amylose content (19–22%) was positively correlated with the 100% rice bread’s specific volume. These authors also identified that the GFB showed smaller bubbles than those derived from low amylose contents, indicating that amylose plays a more important role in achieving a high loaf volume. 

Water is the second most added ingredient in dough production and fundamental for bread’s characteristics and final product [66]. Consistency, flexibility, extensibility, and adhesiveness are entirely or partially determined by water absorption level. In general, the greater the hydration, the higher the specific volume of GFB, until a maximum point at which the dough’s weak structure promotes collapse during the fermentation or baking process [66]. The dough’s hydration level depends on the flour and the characteristics of the final product. On average, 50 to 60% of water is added to wheat flour for protein hydration and gluten formation [112,113]. Our review showed that the studies applied different hydration levels (Table 1) based on previous tests not described in the studies or on previous studies’ formulations.

Water absorption, expressed based on the flour’s weight, refers to the amount of water needed to form a dough of adequate consistency and depends on each formulation. As for the values for water absorption identified in the analyzed studies, we observed a variation between 56 and 130%. Specifically for the studies by Belorio and Gómez [66], Pico et al. [114], and Martínez and Gomez [82], the absorption was 80, 90, and 100%, respectively, probably due to the presence of starch and HPMC in the formulation. In general, GF pasta requires greater hydration than absorption values for wheat-based pasta. The hydration level influences the specific volume; however, the dough’s consistency should not be too low. According to Bravo-Núñez et al. [48], after a certain hydration level, different for each formulation/mass, the specific volume may be lower because there is a value of “limiting consistency” in which the mass is not able to retain air bubbles during cooking, therefore occurring, a drop in volume. Water molecules interact with other compounds, solubilizing the nutrients to yeast development. Its distribution in the dough is decisive for the crumb’s texture characteristics, the crunchiness of the crust, and the bread’s shelf life. Among the factors that affect the formulations’ water absorption capacity are the moisture content, protein content, starch, and other complex carbohydrates, such as the fibers in the formulation [112,113,115]. 

On the role of starch in the performance of formulations for GFB production, the literature states that starches like the ones in corn, potato, cassava, and rice can form a matrix in order to retain carbon dioxide, expand cells, and impair coalescence during growth and to give stabilization for the final structure [27,30,41,116,117]. The choice of starch, therefore, is a crucial element for the proper formulation of such products. Overall, the cereal starches such as wheat, corn, and rice contained a lower moisture level than the tuber starches (potato and tapioca starch). The selected starch type influences the dough’s water absorption and rheological parameters, pasting characteristics of starch, and texture and staling of the obtained crumb [76,118,119].

Many studies have proposed formulations based on a mixture of rice flour, cornflour with starches, hydrocolloids, proteins, and enzymes to obtain GFB with an appropriate specific volume and a crumb with fewer large holes. These substances that improve quality can bind water, increase the viscosity, and develop a non-gluten network. These networks can present gluten properties by stabilized protein bonds (inter and intra). Better dough and improved texture of the products can be achieved with both hydrocolloids and proteins, a mix of viscous properties (protein absorption and flexibility), and elastic properties—film formation and gas retention [14,50,52,53].

It is also essential to consider the properties of the different granules. Rice starch has small size granules (2 to 10 µ), amylose level between 1 to 37%, and when gelatinizes, it has a mild flavor and a creamy appearance, giving a perception of texture similar to that of fat [120]. Corn starch presents 25 to 28% amylose and granules from 5 to 25 µm [121]. Potato starch has amylose levels from 20 to 30% and granule diameter between 20 to 40 µm forming high viscosity, consistent, and clear gel. Cassava starch has 17% of amylose and granule’s size between 30.51–39.50 and 14.39–17.1 µm [122]. 

Gelatinization, an essential technological property for the bakery process, is defined as the collapse of granular ordering. In this irreversible property, changes occur such as granule swelling, crystalline fusion, birefringence loss, granule rupture with the release of amylose, and the increased suspension viscosity. The intensity of hydration and swelling of the starch molecules due to heat, depends mostly on the starch properties as the type of the starch, size, and shape of granules, and the amount of amylose to bind water. The addition of other components to the dough can also change water availability and starch performance [27,30,82].

Belorio and Goméz [66] and Martínez and Goméz [82] proposed starch-based formulations and found close values for hardness (1.44 and 1.25), springiness (1.011 and 0.95), cohesiveness (0.754 and 0.56), and resilience (0.493 and 0.41) of the crumb. Comparing these data with those of Esteller and Lannes [54,55], who evaluated the texture profile of commercial bread made from wheat flour, we found that the values were close to hardness (1.44 and 1.25/1.56), springiness (1.011 and 0.95/0.89), and cohesiveness (0.754 and 0.56/0.67).

Lower values for hardness mean greater softness. The bread’s hardness or firmness is related to the force applied to cause deformation or breakage of the product and is dependent on the formulation. The texture profile data is mainly correlated with the greater incorporation of air in the dough, a greater specific volume of the bread, the resilience, the porosity of the crumb, and the bread’s external characteristics shape and symmetry. In wheat-based doughs, these parameters are related to the constituents’ molecular interactions, such as hydrogen bonds, disulfide bonds, cross-links, and water mobility in the mass [54,55]. 

Twenty-five studies analyzed the parameters of hardness, cohesiveness, chewiness, springiness, and adhesiveness. A wide variation was observed for the hardness parameter and cohesiveness (dimensionless) when compared to the respective control formulations. Cohesiveness refers to the tendency of the molecules to remain together in the matrix. The maintenance of cohesiveness in bread is mainly related to the same components’ molecular interactions that govern volume development [29,32,62,66,76,89,93,95,96,98,101,106,107,108]. 

Springiness refers to the work required to overcome the attractive forces between the material and the probe surface, while chewiness is the energy required to chew the food. The obtained values for springiness varied between 0.95 and 1.03, while the values for chewiness varied between 13.83 and 583.6 N, concerning the respective control formulations. Adhesiveness is the force that the food exerts on the probe preventing it from returning. Only one study evaluated adhesiveness (−4.08). Esteller and Lannes [55] found the following values for cohesiveness (0.67), chewiness (0.94 N.m), springiness (0.89 mm), and adhesiveness (0.001 mJ) for wheat-based loaf bread.

Baked goods’ appearance is influenced by the internal structure and the distribution of gas cells. A proper selection of substances can modify starch systems providing properties such as stabilization, density, gelling, and emulsification. The origin of the starch, but also the non-starchy substances, influence the behavior of these systems. Their stabilization could be influenced by hydrocolloids, surfactants, and other water-soluble molecules [27,30]. 

Starch-based bread showed higher specific volume and lower hardness. The features of wheat starch as building and packing along with its lower pasting temperature create a continuous phase. After gelatinization, a continuous crumb structure may appear [82]. However, Lopez et al. [26] studied the influence of corn and manioc starches and rice flour to produce GFB. They identified that bread differed significantly for a specific volume, crumbly texture, crust’s color, satisfaction level, and outer appearance. Horstmann et al. [76] showed that granule size correlates to baking characteristics. Larger granules are better suited to GFB. 

### 4.1. Hydrocolloids

Regarding the role of hydrocolloids in forming an appropriate matrix for the production of GFB, Baldino et al. [123] reported that these could be the best alternatives to provide gas-retaining and to mimic the viscoelastic properties of gluten. Therefore, they give structure-forming properties to the crumb. The formed network can retain CO_2_, increase loaf volume and improve dough cohesiveness. These agents are also essential to bind water temporarily to gelatinize the starch and structure of the crumb. In gluten-based dough, HPMC addition induces a softening effect but imparts strength to the gelation’s overall network. HPMC interferes with protein chain interactions and aggregation during network formation by replacing the network’s gluten proteins [124]. This phenomenon reinforced micelle integrity during gas expansion, preventing coalescing of the air cells and improving the specific loaf volume of the GFB [102]. 

In formulas of GFB, the addition of hydrocolloids has the objective to improve the cohesive net formation, viscoelasticity, and the capability to hold the formed gas during dough’s fermentation [30,31,37,41]. This influence on GF dough’s rheological properties seems to be related to the polysaccharide’s molecular structure and chain conformation. HPMC structure varies according to the number of methyl groups (non-polar) and hydroxypropyl (polar) inserted in the cellulose polymeric chain. Thus, it affects the polymer polarity and, consequently, the molecule’s ability to interact, especially with proteins or starches [125,126]. Consequently, HPMC improves the gas cells’ stability during expansion, contributing to bread’s specific volume [102,127]. 

The monomers’ spatial distributions and branching are essential to the functional properties of these substances. They strongly influence their behavior when in food matrixes [3,30,31,41,74,128,129]. Branching hydrocolloids act as thickening agents by hydration of the macromolecules. Different hydrocolloid functionality also depends on the interaction with other polymers such as starch and proteins [30,31]. 

In rice flour-based formulations, HPMC can contribute to a matrix with properties to trap carbonic gas, increase the specific volume of the bread, reduce crumble’s hardness, and improve crumbles’ bread structure [29,50,53,54,55,56,57,58]. HPMC contains hydrophilic groups (hydroxypropyl) that produced hydrogen bridges with OH– groups of starch and water. The hydrophobic component (methoxyl groups) works as a surfactant between starch components (amylose and long chains of amylopectin) and the interphase of air cells in the food matrix, reinforcing this structure [49]. Different results were found by Kadan et al. [130] (higher water content, lower specific volume, and a more breakable crumble on the first day of storage). 

In gluten-based dough, HPMC addition induces a softening effect but imparts strength to the gelation’s overall network. HPMC interferes with protein chain interactions and aggregation during network formation by replacing the network’s gluten proteins [124]. This phenomenon reinforced micelle integrity during gas expansion, preventing coalescing of the air cells and improving the specific loaf volume of the GFB [102]. Among the 43 analyzed studies in this systematic review, we found that 49% (*n* = 21) used HPMC in their formulas in a mean concentration equivalent to 2.0%. According to Marco and Rosell [131] and McCarthy [132], HPMC is one of the hydrocolloids most studied and more appropriate to improve the volume and texture of GFB made with rice flour.

Other studies evaluated the addition of XG performance. According to some authors [3,91], XG’s addition improved volume, texture, water retention, and rice bread’s sensory acceptability. XG is an anionic hydrocolloid with two negatively charged carboxyl groups on its side chains. This characteristic favors a higher number of interactions with water molecules, starch, and other XG chain, making it possible to form a rigid matrix to absorb gases (CO_2_). XG in the food matrix contributes to protein-based foams, stable and viscoelastic properties. It may increase loaf volume and improve sensory and rheological properties if we use it in low amounts. 

However, some studies [91,106,133,134,135] identified that the addition of 0.5% of XG increased in small proportions to the rice bread’s volume. Higher levels of XG did not significantly affect the specific volume of bread [3,102]. XG’s addition may not contribute to bread’s volume because it can form a too rigid matrix. Crockett et al. [102] reported that the hydrocolloid performance in GFB depends on the gums’ different chemical structures. Researches recommend XG from 0.5% up to 2.0% (flour weight) in GFB to improve texture [135,136,137]. 

According to Renzetti and Rosell [28], in GFB, HPMC and XG mostly replace gluten in different formulations. During baking, this water-soluble polymer with high surface activity maintains uniformity and stability. No adverse effects on the texture are expected in the final product. Consequently, GFB shows a high specific volume and low crumb hardness [132,138]. 

### 4.2. Enzymes

The use of enzymes in GF batters can improve the breadmaking performance of GF flours. It enables substantial improvement in the gas holding and textural properties of GF batters and bread. The different protein structure type determines enzymatic treatment’s effectiveness, having a specific type of enzymes for each GF system [28]. In gluten-free formulas, the addition of specific enzymes can lead to new chemical links, inner, and intra molecules. It can solubilize insoluble proteins of high molecular weight, strengthen the formula’s net, and improve technological characteristics [30,37,41].

Among the 43 analyzed studies in this research, the enzymes used to compose the formulations were transglutaminase (TGase), Cyclodextrin glycosyltransferase (CGTase), protease, and beta-amylase. TGase is an enzyme that catalyzes an acyl-transfer reaction between the γ-carboxamide group of peptide-bound glutamine residues and various primary amines. According to Moore et al. [139], amino acids and glutamine residues can modify proteins by amine incorporation, cross-linking, and deamination. The CGTase is the only enzyme capable of converting starch and related substances into cyclodextrins through an intramolecular transglycosylation (cyclization) reaction. It also catalyzes intermolecular transglycosylation reactions, such as coupling and disproportionation. Beta-amylase is an exoenzyme that catalyzes the alternate hydrolysis of alpha-1,4-glycosidic bonds of polysaccharides such as starch, releasing maltose molecules from the non-reducing end. The action of the enzyme is interrupted in regions with alpha-1,6-glycosidic bonds. One of its most important properties is thermal lability compared to alpha-amylase [1,113,115]. 

In the analyzed studies, a formulation containing 1.35 UI TGase/g RF proteins and 100% RF produced GFB with a specific volume equal to 5.43 cm^3^/g and 1.2 N for crumb hardness [98]. However, Kringel et al. [79] evaluated the bakery-quality of a formulation containing TGase (1.5 g) and 100% phosphorylated RF and obtained 3.74 cm^3^/g for the specific volume. Borges and Salas-Mellado [68] obtained GFB with a specific volume equal to 3.5 cm^3^/g with a formulation based on 100% RF and 0.1% polysorbate 80. 

Marco and Rosell [131] evaluated the effect of HPMC and TGase in rice-based GFB. They identified a combination of 4% HPMC, 13% soy flour, and 1% TGase decreased the specific volume of the bread and the crumb hardness. Probably, it is justified because TGase reacts differently with various protein sources. 

The study of Gujral et al. [74] was the only one among the selected 43 that evaluated the performance of CGTase (20 µL/100 g) in formulations based on rice flour and HPMC, in different concentrations. The obtained data showed that the highest specific volume of GFB was obtained for the formulation without adding CGTase. Jemli et al. [87] considered that in rice dough, due to the rice proteins’ hydrophobic nature, the use of CGTase could reduce the hydrophobic environment by hydrolyzing and cyclizing the starch [140]. 

### 4.3. Other Additives and Gluten Substitutes

Considering the functionality of food properties, some authors have investigated the effect of supplementation de GFB formulations with egg white, lupine, dairy products, soy flour, and calcium salts at different levels [25,29,30,32,90,93,94,95,96,97,98,99,100,101]. Krupa-Kozak et al. [80] concluded that the supplementation of the formulations with low lactose milk protein products influenced the bread’s quality parameters. It significantly increased the specific volume of all bread; the crust became darker, less rigid, and specific. The addition of milk proteins, soy proteins, GG, pectin, inulin, *Psyllium*, HPMC, or zein to starchy formulations enables to obtain bread with higher specific volume, softer crumble, and better acceptability [64,80,96,101,141,142,143,144,145]. Nevertheless, zein’s addition showed some positive effects on bread quality, but the bread was not acceptable, considering sensory aspects [64,146].

### 4.4. Sensory Analysis and Crust and Crumb Analysis

Bread color is one of the most important indicators of its quality. The desirable crust and color of bread should be golden brown and creamy white, respectively, regarding the crust’s color, and when compared to the studies by Esteller and Lannes [54,55]. Pico et al. [93] found that the L* value (69.79/48.14) was higher, indicating greater light reflectance, which was expressed in the light color of the crust. Likewise, the values of a* (3.08/17.19) and b* (19.34/29.01) also indicated a lighter color for the bread.

Other studies (28%; *n* = 12) determined color of the crumb, crust and both parameters. Values ranged from L* (40.10 to 82.09), a* (2.50 to 5.17) and b* (14.06 to 22.38) for the crust of bread, concerning the control formulation. For the core color, the data obtained were L* (21.77 to 88.96), a* (−1.17 to 5.32) and b* (0.35 to 53.77). Higher positive values for croma a* (redshift) indicate a darker crust. High positive values for croma b* are translated as an intense yellow or golden color, although “diluted” in the brown color characteristic of baked products [55]. The literature reports that the luminosity varies from zero (black) to 100 (white); a* and b* (chromaticity coordinates) range from −a* (green) to + a* (red), while b* values range from +b* (yellow) to −b* (blue) [147]. Esteller [55] reported the following data for crust (L* = 48.14; a* = 17.19; b* = 29.01) and crumb (L* = 62.7; a* = 1.14; b* = 10.88) of wheat-based bread.

The color developed in the dough’s cooking is due to chemical reactions such as pyrolysis, caramelization, and Maillard reaction, or the set of such reactions, depending on each formulation. It is possible to assume that the lower sugar content or the higher starch content in the formulation contributed to the crust’s light color obtained for these products [54]. 

Three studies (9%) performed the sensory analysis of the products. Nishita et al. [84] performed the analysis with 57 tasters who classified GFB as “slightly disliked”, with a mean value of 4.2 on a nine-point scale. Crockett et al. [102] presented the following data with 28 tasters: average score in acceptability testing equal to a 4.0 ± 2.0; texture: too dry, coarse, sponge-like, sandy, foamy, beany flavor, chemical aftertaste, and appearance shine is unappealing. Graça et al. [72] obtained a higher Acceptance Index (75%) in a formulation with 4% collagen.

Four studies carried out image analysis of the cells of the kernels. They identified the following results: (1) that the GFB obtained from a formulation based on unripe banana flour (specific volume = 4.82 cm^3^/g) showed that 0.95% of the cells were large, the number of the large cell was 6.17, whereas the number of the largest cell was 1.47; (2) that the GFP obtained from a formulation based on RF/swett potato flour (PF)/buckwheat flour (BCWF) (specific volume = 4.52 cm^3^/g) showed mean cell area (mm^2^) equal to 0.339, circularity equal to 0.699 mm and cell density equal to 811, 21; (3) that the GFB obtained from a PS-based formulation added of 12% sodium caseinate (specific volume = 3.56 cm^3^/g) showed porosity equal to 0.409, cell density (1/cm^2^) equal to 9.0 and % of pores > 5 mm equal to 0.446; (4) that the GFB obtained from a formulation based on MS/potato starch (PS)/red potatoes showed porosity equal to 0.401, number of rotten equal to 1408, number of pores/cm^2^ equal to 4779 (Table 1) [49,73,97,148]. Esteller [55] found for the porosity of the bread crumb of the commercial loaf, based on wheat flour, an average area of 0.41 mm^2^, half diameter equal to 0.38 mm, and average perimeter equal to 1.37 mm.

Porosity refers to the alveolar structure of the loaves. The number and volume of alveoli are directly related to the formulation and the baking processes. Masses with a large amount of liquids (greater water absorption) tend to have crumbs with large dimensions (wide and deep) identified in the average area’s values. As loaves of bread are obtained through a cylindrical dough, they tend to form products with a more homogeneous crumb, a greater number of alveoli, and smaller volumes [55].

In breadmaking, the performance of ingredients such as starches, proteins, enzymes, among others, has already been extensively studied and understood, differently from what occurs with the production of bread with mixed flours, or specifically, formulations for the production of GFB. For Horstmann et al. [76], studies should be conducted to identify interactions between different components and their behavior in a model bread system. 

Among the 43 selected studies for this systematic review, we found that 9 (21%) did not use flour; 25 (58%) used rice flour; 7 (16%) used rice flour with other flours (sweet potato, maize, buckwheat, soy, chestnut, locust bean, and cassava) [65,82,89,91,96,97,99]. Eighteen (42%) used corn, and 14 32%) used potato starch, and that HPMC, among hydrocolloids, was the most used (*n* = 24; 56%). It is suggested, therefore, that from the information obtained in this systematic review, and according to Horstmann et al. [31], further investigations need to be conducted based on the formulations proposed by these 43 studies, looking for a deeper understanding of gluten-free systems that could help to gain a fundamental understanding of how GF ingredients can replace wheat flour. According to Martínez and Goméz [82], rice flour is the most commonly used (as found in our review), followed by maize flour. 

### 4.5. Risk of Bias (RB) 

Bias risk evaluation is fundamental to evaluate a study’s quality once it is directly related to two dimensions: inner and outer validity. Internal validity answers the study question appropriately, free of bias. It is determined by how the design, data gathering, and analysis were conducted and exposed to bias. The external validity is related to the study question, and if it was created, it can generalize and apply the results in other scenarios [149].

Of the 43 articles with a specific volume greater than 3.5 mL/g, 3 had no risk of bias [72,83,90], because all met the requirements for their assessment: (1) characterization of the raw material, ingredients, and gluten substitutes; (2) physical characteristics of the bread; (3) sensorial analysis of the product; (4) function of each ingredient; (5) experimental design; (6) statistical test; (7) results answer the main question. Among articles with a specific volume greater than 3.5 cm^3^/g, 67% (*n* = 29) had a low risk of bias, and 26% (*n* = 11) of studies with a specific volume greater than 3.5 cm^3^/g had a high risk of bias.

### 4.6. Limitations

Some methodological limitations of this review should be highlighted: (1) the inner and outer characteristics of GFB such as hardness, springiness, cohesiveness, crust and crumb colors, crumb’s porosity, specific volume were evaluated only in 51% (*n* = 22) of the studies; (2) different parameters to evaluate the texture profile of the bread, as well as different ways of expressing the results; (3) only 12% (*n* = 5) of the studies included sensory tests; (4) sensory analysis was conducted with a small number of tasters; (5) only 23% (*n* = 10) determined or the crust color or the crumb color or both colors; (6) only 9% (*n* = 4) analyzed the characteristics of the alveolar structure of the GFB core; (7) absence of an identity standard and quality of GFB formulations to compare the results obtained; (8) Some studies (*n* = 9) used laser sensor with the Volscan Profiler (StablE Micro Systems, Godalming, UK) [46,48,66,76,77,78,94,114] and the others used AACC method 10–05 [150]. In the analysis technique using the AACC method, as it is a subjective measure, human error may be considered an accepted and validated method for this type of analysis [150]. The use of the scanner technique is less prone to errors for using equipment to measure volume. However, both assess bread volume with results measured in cm3/g, allowing comparison between results.

Another important limitation is the fact that we have not evaluated the changes that occur during product storage. In starch-based GFB, it is also essential to consider that significant causes of the bread firming are starch retrogradation, recrystallization of amylose, and amylopectin formation as the gelatinized starch cools [65]. The phenomenon significantly impacts other food products’ characteristics. Reconnection of amylopectin molecules via hydrogen bonds leads to texture changes of starch gels, even those highly concentrated such as bread, especially during their initial storage—24 h after preparation [30]. The bread crumb resistance to deformation is the textural attribute referred to as firmness and is an essential factor in staling. The degree of firmness and increase in crumbliness is commonly used to assess bread’s staling and an essential staling indication [130,151,152].

Other ingredients like sugar, yeast, salt, oil, and their effect on bread volume are important in gluten-free breadmaking. However, they were not discussed in this review since they are common ingredients on bread and GFB formulations. Considering the study on GFB, there are several variations in the use of starch sources, gluten substitutes, and additives, the focus of our discussion. However, we recognize the importance of discussing the other ingredients and their amounts used in GFB formulations in further studies to achieve the GFB best formulations.

## 5. Conclusions

Our hypothesis considered the specific volume greater than or equal to 3.5 cm^3^/g as a bread quality predictor. Thus, of the 259 studies analyzed in this systematic review, 43 proposed formulations producing GFB with a specific volume greater than or equal to 3.5 cm^3^/g.

In general, the results showed that rice and corn flours are the most studied considering GFB specific volume ≥ 3.5 cm^3^/g. Based on specific volume parameter, the formulations that showed the best technological aptitude used as primary starch sources the rice flour, rice flour plus corn starch, or corn starch plus rice starch. Also, most of them used 2% HPMC. However, it is necessary to jointly analyze other parameters that contribute to the quality, such as texture profile, external and internal characteristics, acceptability, and useful life of the products, especially since it is a product obtained through raw materials and unconventional ingredients. As pointed out in this review, specific volume by itself does not guarantee the production of GFB without large holes or compact structures, nor it guarantees acceptable sensory characteristics. GFB production is challenging because it deals with the choice of the main ingredients and additives to replace gluten and the interaction of common bread´s ingredients inside the new formulations.

In this selection, no studies were found with formulations with other flours such as sorghum, pseudocereals, corn, soy, chestnut, cassava, pine nut, teff, millet, acorn, and technologies such as high hydrostatic pressure, sourdough, among other unconventional ones. 

According to some of the limitations pointed out by us, it is also essential to consider creating an identity and quality standard for gluten-free bread and formulations to make comparisons with an official standard, as with wheat-based bread. It is important to note that, as the specific volume, the texture profile analysis (hardness, chewiness, springiness, cohesiveness, and adhesiveness) is a predictor of GFB quality. Specific volume is an important quality indicator, but to obtain specific volumes greater than or equal to 3.5 cm^3^/g on GFB, improving additives are often artificial and not always tolerated by consumers. Therefore, it is important to look for GFB formulations based on natural additives, which could be the subject of another review.

## Figures and Tables

**Table 1:** Extraction table containing references, starch sources, gluten substitutes, additives, water, specific volume, crumb and crust characteristics, and sensory analysis of gluten-free bread with specific volume above 3.5 cm^3^/g.

Author (Year)	Starch Sources, Gluten Substitutes, and Additives	Water (% of Flour Weight)	Best Formulation (% of Flour Weight)	Specific Volume (cm^3^/g)	Crumb and Crust	Sensory Analysis
Andersson et al. (2011) [64]	Zein, CS, HPMC, β-Glucan, MS, OW28.	64 to 80%	Flour (20% zein + 80% CS) + 2% HPMC + 2% salt + 5% sugar + 1% yeast + 75% water	4.4	-	-
Aoki (2018) [65]	RF. Formulations in which 1, 2, and 5% of the rice flour were replaced with sweet potato flour and β-amylase.	90%	Flour (100% RF) +2% salt + 9% sugar + 2% yeast + 2.5 olive oil 90% water	4.20	Firmness (N) = 0.55	-
-	-	90%	Flour (100% RF—Mizuhockikara with 22.3% of amylose) + 0.00005% protease A+ 2% salt + 9% sugar + 2% yeast + 2.5 olive oil 90% water	5.0		
Belorio & Gómez (2020) [66]	RF or MS and hydrocolloid (HPMC, XG or *Psyllium*)	70 to 120%	Flour (100% MS) + 2% HPMC + 6% sunflower oil + 5% sugar + 3% yeast power + 1.8 salt + 80% water	7.58	Hardness (N) = 1.44 Springiness = 1.011 Cohesiveness = 0.754 Resilience = 0.493	-
Berta et al. (2019) [67]	CS, PS, zein and HPMC	39.4%	Flour (86% CS + 14% PS) + 5% zein + 5% HPMC + 3% salt + 4% sugar + 2% dry yeast + 6% olive oil + 82% water	6.0	Firmness (N) = 5.5 Cohesiveness = 0.70 Crust hardness (N)~3	-
Borges & Salas-Mellado (2016) [68]	RF, MC and TGase added by sorbitol, trehalose, alpha-amylase, or polysorbate	75%	Flour (100%RF) + 2% MC + 6% vegetable oil + 5% sugar + 2% yeast + 2% salt + 75% water+ 0.1% polysorbate + 1% vegetable oil + 0.5% TGase + 0.0009% ascorbic acid	3.5	Hardness (N) = 3.07	-
Bravo-Núñez et al. (2019) [48]	MS, and HPMC added by pea protein and/or EWP	80.49 to 139.22%	Flour (70% MS + 30% EWP) + 2% HPMC + 6% oil + 5% sugar + 3% yeast + 1.8% salt + 80.49% water	5.5	Hardness = 21.98 Springiness = 1.03 Cohesiveness = 0.61 Chewiness = 13.83	-
Chakraborty et al. (2016) [69]	Millet and soybean flour	80%	Flour (85% millet flour + 15% soy flour) + 2.6% salt + 3% sugar + 5% yeast + 80% water	3.44	Hardness = 159.1N Cohesiveness = 0.77 Resilience = 0.64 Springiness = 0.98 L* = 21.77; a* = 117.54 b* = 98.87	-
Crockett et al. (2011) [70]	RF and CS (added or not with Methocel E15-HPMC) + soy protein and/or EWP.	132 to 148%	Flour (67% RF + 33% cassava starch) + 16% HPMC + 50% EW + 10% yeast + 2% salt + 4% sugar + 148% water	~4.0	Hardness (N)~8.0 Springiness~8.0	N = 28 The average score in acceptability testing 4.0 ± 2.0 Texture: too dry, coarse, sponge-like, sandy, foamy Flavor: beany, chemical aftertaste
de la Barca et al. (2010) [71]	Popped AF and raw AF	58%	Flour (70% popped AF + 30% raw AF) + 2% yeast + 2% salt + 6% sugar + 58% water	3.5	-	-
Graça et al. (2017) [72]	RF, ascorbic acid, MC, TGase, collagen powder and collagen fiber. Control: without collagen powder or collagen fiber.	120%	Flour (100% RF) + 2% MC + 0.5% TGase + 4% collagen fiber + 2% salt + 5% sugar + 2% dry yeast + 6% soy oil + 0.009% ascorbic acid + 120% water	3.8	Crumb firmness~0.2 Color crust L* = 74.00; a* = 2.50 b* 22.38 Color crumb L* = 70.40; a* = −1.02 b* 6.75	80 tasters Sensory acceptance 75%
Gumul et al. (2017) [73]	Control: MS, PSGG. GFB+5BS: Control+ freeze-dried red potatoes (Blue Star variety). GFB+5ML: Control+ freeze-dried red potatoes (Magenta Love variety). GFB+SV: Control+ freeze dried red potatoes (Violeta variety).	103%	Flour (80% MS + 20% PS -Magenta love variety) + 1.7% GG + 1.7% pectin + 5% yeast + 1.7% salt + 2% sucrose + 3% oil + 103% water	3.56 mL/g	Number of pores = 1408 Porosity (%) = 0.401 Number of pores/cm^2^ = 4779	
Gujral et al. (2003) [74]	RF, HPMC, and CGTase.	90%	Flour (100% RF) + 0.00002% CGTase + 4% HPMC + 6% oil + 7.5% sugar + 2% salt + 3% yeast + 90% water	4.3	Crumb firmness = 247.1 g	
Han et al. (2019) [75]	Control: flour (a mixture of garbanzo bean flour, PS, TF, whole grain sorghum flour and fava bean flour), rice fiber, TS. M5: mix flour, rice fiber, 6 g TS, white egg M200. M10: mix flour, rice fiber, TS, trehalose, white egg M200. M15: mix flour, rice fiber, tapioca starch, trehalose, and white egg M200. P5: mix flour, rice fiber, TS, trehalose, white egg P110. P10: mix flour, rice fiber, TS, trehalose, soybean oil, white egg P110. P15: mix flour, rice fiber, TS, sugar, trehalose, soybean oil, white egg P110.	100%	M15: Flour (85% mix flour + 15% egg white solid) + 4% rice fiber+ 3% TS + 10% sugar + 1.6% salt + 5% trehalose + 12% soybean oil + 3% yeast +100% water + 15% EW M200.	4.45	Hardness (N) = 5.1 Springiness = 0.95 Cohesiveness = 0.78 Chewiness = 3.7 Resilience = 0.46	-
Hernández-Aguirre et al. (2019) [49]	Unripe banana flour(UBF), HPMC, Pregelatinized Unripe banana flour(UBF-P)	46 to 100%	Flour (75% UBF + 25% UBF-P) + 4% HPMC + 73% fresh eggs + 8% sugar, 8% shortening, 1% instant yeast + 2% salt	4.82	Large cell (%) = 0.95 Number of large cells 6.17 Number of the largest cell 1.47	-
Horstmann et al. (2016) [76]	WS, PS, TS, MS, RS, HPMC.	80%	Flour (100% PS) + 2% HPMC + 2% salt + 4% sugar + 2% yeast + 80% water	5.0	-	-
Horstmann et al. (2017) [77]	PS, HPMC and protein source (potato protein, soy protein isolate, pea protein lupin protein, carob protein)	80%	Flour (100% PS) + 2% HPMC + 2% lupin protein + 2% salt + 4% sugar + 2% yeast + 80% water	3.66	Hardness (N) = 7.12 Springiness rate = 0.230 Cohesiveness = 0.057 Resilience = 0.049	-
Horstmann et al. (2018) [78]	PS, and hydrocolloid (GG/XG/LGB/HPMC/pectin/sodium alginate)	80%	Flour (100% PS) + 1% sodium alginate + 2% salt + 4% sugar + 2% yeast + 80% water	3.6	Hardness (N) = 4.3	-
Kringel et al. (2017) [79]	RF with native RF or phosphorylated RF, ascorbic acid, MC, and TGase.	120%	Flour (100% RF phosphorylated) + 2%MC + 0.5% TGase + 2% salt + 2% soy oil + 2% dry yeast + 5% sugar + 120% water	3.74	Color crust L* = 70.12; a* = 4.02 b* 26.08 Color crumb L* = 74.21; a* = −0.82 b* 9.35	-
Krupa-Kozak et al. (2013) [80]	PS; P; Calcium citrate; MS added by protein Calcium caseinate, sodium caseinate, isolated whey protein, and hydrolyzed whey proteins)	105%	Flour (79% CS + 21% PS) + 5% pectin + 2% salt + 6% sugar + 6% dried yeast + 3% oil + 8% calcium citrate + 16% sodium caseinate + 105% water	4.7	Hardness (N) = 11.43 Springiness = 0.981 Cohesiveness = 0.427 Chewiness = 475.84 Crust L* = 30.25; a* = 8.31 b* = 13.02 Crumb L* = 69.92; a* = −0.95 b* = 12.55	-
Mancebo et al. (2017) [81]	RF and HPMC added by different percentages of oil	70 to 100%	Flour (100% RF) + 2% HPMC + 5% sucrose + 1.8% salt + 3% instant yeast + 20% oil + 100%	4.0	Highest value for the a* and b* parameters of the crust. It also decreases hardness, cohesiveness, springiness, and the L*	-
Martinez & Gomez (2017) [82]	MF/RF, MF/MS, MF/PS and HPMC.	100%	Flour (100% MF/MS) + 2% HPMC + 3% instant dry yeast + 6% oil + 1.8% salt + 5% white sugar	7.14	Hardness (N) = 1.25 Springiness = 0.95 Cohesiveness = 0.56 Resilience = 0.41 Crust color L* = 82.09; a* = 2.64 b* = 19.32	-
Matos & Rosell (2013) [83]	Gluten-free commercial mixture; or RF + HPMC; or RF +MS + PS+ soy protein + XG; or RF + MS + PS + pectin; or RF + MS + PS+ skim milk powder + whole egg powder + XG + HPMC; or RF + PS + skim milk powder + HPMC.	56.5 to 120%	Flour (50% RF + 50% PS) + 2.2 HPMC + 5% fresh yeast + 5% sugar + 6% vegetable oil + 2% salt + 10% skim milk powder + 79% water	5.07	Hardness (N) = 5.43 Crumb color L* = 81.50; a* = −1.53 b* = 6.475 -	GFB Crumb appearance 3.17 Taste = 3.33 Odor = 2.83 Springiness 2.33 Hardness (N) 4.33 Crumbiness 3.00
Nishita et al. (1976) [84]	RF, + MC/GG/LBG/CMC-Na/XG/DG/SSL2/CSL2/SMG/EMG.	75%	Flour (100% RF) + 3% of MC–90 HG 4000 + 3% compressed yeast + 7.5% sugar + 6% vegetable oil + 2% salt + 75% water	5–5.3	Very good crumb, white coloring, satisfactory flavor, when fresh.	57 tasters—“slightly disliked” bread (4.2 on a 0–9 scale)
Nishita & Bean (1979) [85]	RF + MC.	75%	Flour (100% RF) + 3% of MC-90 HG 4000 + 3% compressed yeast + 7.5% sugar + 6% vegetable oil + 2% salt + 75% water	5.2–5.7	Good crumb texture.	-
Olojede et al. (2020) [86]	SF + cowpea flour. + Sourdough (*Pediococcus pentosaceus* SA8).	105%	Flour (90% SF + 10% cowpea flour) + 2% salt + 4% sugar + 1% baking fat + 2% compressed yeast.	3.63	Hardness (N) = 26.40 Cohesiveness = 0.21 Springiness = 10.93 Gumminess = 0.56 Chewiness = 6.14 Crust color L* = 40.10; a* = 5.17 b* = 14.06 Crumb color L* = 42.78; a* = 5.32; b* = 13.99	Appearance (7.09) Taste (7.09) Texture (7.82) Aroma (7.09) Crumb (7.45) (Scale 0–9)
Ozturk & Mert (2018) [87]	CGM + CS + GG or HPMC.	83.33%	Flour (22% CGM + 78% CS) + 5% HPMC + 1% dry yeast + 5% sugar + 2% salt + 83.33% water	3.46	Microfluidization and the addition of HPMC decreased hardness and increased springiness and cohesiveness. L* = 88.96; a* = 3.00 b* = 53.77	-
Ozturk & Mert (2018) [88]	CGM + CS+ XG or citrus fiber	75 to 93.75%	Flour (22% CGM + 78% CS) + 5% XG + 1% dry yeast + 5% sugar + 2% salt + 83.33% water	3.59	Lower hardness, higher cohesiveness, and springiness values were obtained as a result of microfluidization and supplement addition.	-
Paciulli et al. (2016) [89]	F1: MS, PS, skimmed milk, dextrose, cellulose, GG and HPMC. F2: MS, RF, lupine proteins, dextrose, HPMC, vegetable fiber. Control: commercial mixture.	88 to 90%	Flour (43.5% CS + 40% RF+ 6.5% lupine proteins) + 4.5% destrose + 2% HPMC + 2% vegetable fiber + 3.5% salt + 5% yeast + 5% sunflower oil + 90% water	5.1	Crumb Hardness (N) = 0.70 Cohesiveness—0.81 Resilience—0.44 Chewiness (N)—0.50 Crust color L* 77.5; a* 2.8; b* 17.1 Crumb color L* 76.1; a* 1.5; b* 11.8	-
Pasqualone et al. (2010) [90]	Control: MS; Cassava bread with oil (CBO): CS; Cassava bread with EWP (CBE): CF + EW; Cassava bread with EWP and extra-virgin olive oil (CBOE): CF + EWP.	100 to 120%	Flour (100% cassava flour) + 2.5% fresh compressed yeast + 9% sucrose + 2% salt + 6% extra virgin olive oil + 40% EWP + 100% water	3.93	Crumb color = 3.3 Crust thickness = 1.6 Crumb Firmness (N) = 4.67 Cohesiveness = 7.3 Consistency = 4.7 Overall acceptability = 8.4	-
Peressini et al. (2011) [91]	RF, BWF, salt, and XG or PGA.	80 to 100%	Flour (60% RF + 40% BWF) + 1.5% salt + 4.4% oil + 5.3% compressed yeast + 1.5% PGA	3.78	Firmness (N) < 2	-
Pérez-Quirce et al. (2014) [92]	RF and HPMC-SFE or BG.	70 to 110%	Flour (100% RF) + 6% oil + 5% sucrose + 2% salt + 3% dried yeast + 1.6% HPMC-SFE + 90% water	4.80	Firmness (N) = 1.0 Chewiness = 0.27 Resilience = 0.22 Cohesiveness = 0.47 Springiness = 0.57 Crumb color L* = 73; h = 90; C* = 7 Crust color L* = 61; h = 65; C* = 31	-
Pico et al. (2019) [93]	Control: RF, HPMC and MS + protein source (rice protein, pea protein, egg protein, or whey protein)	90%	Flour (95% RF + 5% rice protein) + 2% HPMC + 5% sucrose + 1.8% salt + 3% instant yeast + 6% sunflower oil + 90% water	7.58	Crust thickness 3.99 ± 0.16 Crust color L* 69.79; a* 3.08 b* 19.34	-
Roman et al. (2019) [94]	MS, RS, HPMC, native banana starch (NB), and extruded banana starch.	105%	Flour (80% MS and RF + 20% NB) + 2% HPMC + 5% white sugar + 2% salt + 3% instant yeast + 6% oil + 105% water	5.34	Hardness (N) = 3.04 Springiness = 0.99 Cohesiveness = 0.45 Resilience = 0.21	Appearance 6.5 Odor 6.0 Flavor 5.3 Texture 5.8 Overall liking 6.0
Roman et al. (2019) [40]	Flour (waxy rice flour, basmati rice flour, Thai rice flour, sushi rice flour or bomba rice flour) and HPMC	90%	Flour (100% Bomba rice flour) + 2% HPMC + 5% white sugar + 2% salt + 3% instant yeast + 6% oil + 90% water	4.85	Hardness (N) = 0.88 Cohesiveness = 0.67 Resilience = 0.32	-
Roman et al. (2020) [46]	MS, RF, and HPMC added with Acetylated di-starch adipate (ADA), Di-starch phosphate (DP), and/or Pre-gelatinized acetylated di-starchphosphate (PADP) starches.	120%	Flour (90% RF + 10% DP) + 2% HPMC + 5% white sugar + 2% salt + 3% instant yeast + 6% oil + 90% water	5.08	-	-
Sahagún et al. (2020) [95]	MS +HPMC added with pea or EWP	90%	Flour (100% MS + 69% EWP) + 2% HPMC + 5% sugar + 1.8% salt + 3% yeast + 6% oil + 90% water	5.47	Hardness (N) = 22.34 Springiness = 1.00 Cohesiveness = 0.61 Chewiness = 13.61	-
Sánchez et al. (2004) [96]	MS, RF, CS, soy flour and milk powder	83.33%	Flour (74.2% MS + 17.2% RF + 8.6% CS) + 7.5% soy flour + 7.8% dry milk + 10% fat + 3% HPMC + 5% sugar + 3% salt + 10% yeast + 83.33% water	3.7	Crumb grain score = 7.9 Bread score = 74	-
Southgate et al. (2017) [97]	RF + sweet cassava flour + BWF	100%	Flour (30% RF + 25% sweet CF + 45% BWF) + 3% sugar + 2% salt + 1.5% instant active dry yeast + 6% vegetable oil + 100% water	4.52	Mean cell area (mm^2^) 0.339 Cell density = 811.21 Circularity = 0.699	Sensory acceptability analysis = 6.56 (0 to 9)
Storck et al. (2013) [98]	RF, flour improver, XG and TGase (egg albumin and casein	115%	Flour (100% RF) + 1.35UI TGase + 0.67% albumin + 5% sugar + 3% salt + 2% compressed yeast + 3% soy oil + 1% XG + 3% bread improver + 115% water	5.43	Hardness (N) = 1.2 Adhesiveness = −4.08 Cohesiveness = 0.51 Chewiness = 583.6	-
Tsatsaragkou et al. (2017) [99]	RF, carob flour, EWP, WP, shortening, DATEM, LBG and enzyme. Commercial mixtures: (C1): a mixture of RF, MF, and PS (C2): wheat starch, sugar beet fiber, HPMC, and GG. (C3): CS, RF, BWF, dextrose, and thickeners (carob seeds, C, HPMC).	80 to 120%	Flour (100% C3) + 6% fresh yeast + 3.5% shortening + 3% sugar + 2% salt + 85% water	3.95	Lower crumb firmness values and acceptable elasticity and porosity.	-
Yano et al. (2017) [100]	RF (low starch damage).	87.5%	Flour (100% RF—low starch damage <5 g/100 g) + 9.3% sugar + 1% salt + 3.1% yeast + 1.2% butter+ 87.5% water	4.0	-	-
Ziobro et al. (2013b) [101]	CS, PS, GG, P + protein source (albumin, lupine protein, soy protein concentrate, pea, and collagen)	130%	Flour (72% CS + 18% PS) + 10% albumin + 1.7% GG + 1.7% pectin + 5% yeast + 1.7% salt + 2% sucrose + 3% oil + 130% water	4.7	Porosity = 0.409 Cell density (1/cm^2^) = 9.0 % de pores > 5 mm = 0.446 Hardness (N) <1.0 Cohesiveness~1.0 Chewiness <1.0 Crumb L* = 84.68; a* = −1.17 b* = 14.40	

AF—Amaranth Flour; AM—Amylase; AMY—Maltogenic amylase; AXs—Arabinoxylans; BG—Beta-glucan; BWF—Buckwheat flour; C—Carrageenan; CGM—Corn Gluten Meal; CGTase—Cyclodextrin glycosyltransferase; CMC—Carboxymethylcellulose; CMC+Na—Sodium carboxymethylcellulose; CF—cassava flour; CSL—Calcium stearoyl-2-lactylate; CS—Corn Starch; DATEM—Diacetyl tartaric acid ester of monoglycerides; DF—Dietary fiber; EMG—Ethoxylated monoglycerides; EW—Egg White; EWP- Egg White Protein; FWB—flour weight basis; GF—Gluten-free; GFB—Gluten-free bread; GG—Guar gum; GMS—Glyceryl monostearate; GO—Glucose oxidase; HG—Methocel series; HPC—Hydroxypropylcellulose; HPMC—Hydroxypropylmethylcellulose; HPMC-SFE—Hydroxypropylmethylcellulose semi-firm; KG—Konjac gum; LAB—Lactic acid bacteria’s; LBG—Locust bean gum/Carob gum; MC—Methylcellulose; MCC—Microcrystalline cellulose; MF—Maize flour; MFAX—Maize fiber arabinoxylans; MS—Maize starch; OW28—Oatwell^®^; PS—Potato Starch; PGA—Propylene glycol alginate; RS—Resistant Starch; RF—Rice flour; SBF—Sugar beet fibers; SF—sorghum flour; SMG—Succinylated monoglycerides; SSL—Sodium stearoyl lactylate; TS—Tapioca starch; TGase—Transglutaminase; XG—Xanthan gum.

## Data Availability

The study did not report any data.

## Supplementary Materials

The following are available online at https://www.mdpi.com/2304-8158/10/3/614/s1, Table S1: Database search strategy.
