# Supplementary material for: A Systematic Review on Gluten-Free Bread Formulations Using Specific Volume as a Quality Indicator
Source: Foods. 2021 Mar 13;10(3):614. doi: 10.3390/foods10030614 (PMC7999268; doi:10.3390/foods10030614)
Supplement: Supplementary file 1 [file foods-10-00614-s001.pdf]

Table S1: Database search strategy.

Database	Search (Jun 1st, 2016, Feb 22th, 2019 and Jan, 2nd, 2021)
**Science Direct**	("bakery products" OR "bakery product" OR breadmaking OR bread OR bread*) **AND** ("gluten free" OR "gluten replacers" OR "without gluten" OR "gluten free flours" OR "gluten free flour" OR maize* OR "maize zein" OR zein* OR rice* OR "rice flour" OR "rice starch" OR sorghum* pseudocereal* OR quinoa* OR amaranth* OR buckwheat* OR starch* OR corn* OR "corn flour" OR "corn starch" OR potato* OR "potato starch" OR manioc* OR "manioc flour" OR "cassava flour" OR cassava* OR "cassava starch" OR tapioca OR yam* OR "legume flour" OR "legume flours" OR soya* OR soy* OR carob* OR vinal* OR bean* OR lentil* OR chickpea* OR pea* OR psyllium OR hydrocolloids OR hydrocolloid* OR emulsifier* OR cellulose* OR carboxymethylcellulose OR hydroxypropylmethylcellulose OR methylcellulose OR guar* OR xanthan* OR alginate* OR "Guar gum" OR "Xanthan gum" OR "green banana" OR "unripe banana" OR "unripe plantain" OR "organic banana" OR "green plantain" OR cavendish OR banana OR plantain OR "green banana's biomass" OR "green banana's flour" OR "green banana flour" OR "flour of green banana" OR "biomass of green banana" OR "green banana biomass" OR "green banana's biomass" OR "unripe plantain flour" OR "unripe plantain's flour" OR "flour of unripe plantain" OR "unripe plantain biomass" OR "biomass four unripe plantain" OR "unripe plantain's biomass" OR "banana flour" OR "Banana pulp flour" OR "Banana peel flour" OR "banana pulp" OR "Tragacanth gum" OR "Karaya gum" OR "Gellan gum" OR agar OR "Locust bean gum" OR carrageenan* OR pectin* OR flaxseed* OR "Chia seed" OR linseed* OR seed* yacon* OR millet* OR chestnut* OR "teff flour" OR teff* OR "high-ester pectin" OR fructooligosaccharide OR inulin* OR "Oat ß-glucan" OR agarose* OR "gluco-oligosaccharide" OR "lupine fiber") **AND** (food OR "food science" OR nutrition* OR technological)
**Scopus**	("bakery products" OR "bakery product" OR breadmaking OR bread OR bread*) **AND** ("gluten free" OR "gluten replacers" OR "without gluten" OR "gluten free flours" OR "gluten free flour" OR maize* OR "maize zein" OR zein* OR rice* OR "rice flour" OR "rice starch" OR sorghum* pseudocereal* OR quinoa* OR amaranth* OR buckwheat* OR starch* OR corn* OR "corn flour" OR "corn starch" OR potato* OR "potato starch" OR manioc* OR "manioc flour" OR "cassava flour" OR cassava* OR "cassava starch" OR tapioca OR yam* OR "legume flour" OR "legume flours" OR soya* OR soy* OR carob* OR vinal* OR bean* OR lentil* OR chickpea* OR pea* OR psyllium OR hydrocolloids OR hydrocolloid* OR emulsifier* OR cellulose* OR carboxymethylcellulose OR hydroxypropylmethylcellulose OR methylcellulose OR guar* OR xanthan* OR alginate* OR "Guar gum" OR "Xanthan gum" OR "green banana" OR "unripe banana" OR "unripe plantain" OR "organic banana" OR "green plantain" OR cavendish OR banana OR plantain OR "green banana's biomass" OR "green banana's flour" OR "green banana flour" OR "flour of green banana" OR "biomass of green banana" OR "green banana biomass" OR "green banana's biomass" OR "unripe plantain flour" OR "unripe plantain's flour" OR "flour of unripe plantain" OR "unripe plantain biomass" OR "biomass four unripe plantain" OR "unripe plantain's biomass" OR "banana flour" OR "Banana pulp flour" OR "Banana peel flour" OR "banana pulp" OR "Tragacanth gum" OR "Karaya gum" OR "Gellan gum" OR agar OR "Locust bean gum" OR carrageenan* OR pectin* OR flaxseed* OR "Chia seed" OR linseed* OR seed* yacon* OR millet* OR chestnut* OR "teff flour" OR teff* OR "high-ester pectin" OR fructooligosaccharide OR inulin* OR "Oat ß-glucan" OR agarose* OR "gluco-oligosaccharide" OR "lupine fiber") **AND** (food OR "food science" OR nutrition* OR technological) **AND NOT** (cake* OR biscuit* OR brownie* OR danish OR "cinnamon Roll" OR cookie* OR croissant* OR donut OR muffin* OR pasta OR beverage OR beverages OR cracker OR snacks OR extruded OR dessert* OR meat OR geography OR history OR philosophy OR anthropology OR economy OR animals OR insects)
**Sprigerlink**	("bakery products" OR "bakery product" OR breadmaking OR bread OR bread*) **AND** ("gluten free" OR "gluten replacers" OR "without gluten" OR "gluten free flours" OR "gluten free flour" OR maize* OR "maize zein" OR zein* OR rice* OR "rice flour" OR "rice starch" OR sorghum* pseudocereal* OR quinoa* OR amaranth* OR buckwheat* OR starch* OR corn* OR "corn flour" OR "corn starch" OR potato* OR

	"potato starch" OR manioc* OR "manioc flour" OR "cassava flour" OR cassava* OR "cassava starch" OR tapioca OR yam* OR "legume flour" OR "legume flours" OR soya* OR soy* OR carob* OR vinal* OR bean* OR lentil* OR chickpea* OR pea* OR psyllium OR hydrocolloids OR hydrocolloid* OR emulsifier* OR cellulose* OR carboxymethylcellulose OR hydroxypropylmethylcellulose OR methylcellulose OR guar* OR xanthan* OR alginate* OR "Guar gum" OR "Xanthan gum" OR "green banana" OR "unripe banana" OR "unripe plantain" OR "organic banana" OR "green plantain" OR cavendish OR banana OR plantain OR "green banana's biomass" OR "green banana's flour" OR "green banana flour" OR "flour of green banana" OR "biomass of green banana" OR "green banana biomass" OR "green banana's biomass" OR "unripe plantain flour" OR "unripe plantain's flour" OR "flour of unripe plantain" OR "unripe plantain biomass" OR "biomass four unripe plantain" OR "unripe plantain's biomass" OR "banana flour" OR "Banana pulp flour" OR "Banana peel flour" OR "banana pulp" OR "Tragacanth gum" OR "Karaya gum" OR "Gellan gum" OR agar OR "Locust bean gum" OR carrageenan* OR pectin* OR flaxseed* OR "Chia seed" OR linseed* OR seed* yacon* OR millet* OR chestnut* OR "teff flour" OR teff* OR "high-ester pectin" OR fructooligosaccharide OR inulin* OR "Oat ß-glucan" OR agarose* OR "gluco-oligosaccharide" OR "lupine fiber")<br>**AND** (food OR "food science" OR nutrition* OR technological)<br>**NOT** (cake* OR biscuit* OR brownie* OR danish OR "cinnamon Roll" OR cookie* OR croissant* OR donut OR muffin* OR pasta OR beverage OR beverages OR cracker OR snacks OR extruded OR dessert* OR meat OR geography OR history OR philosophy OR anthropology OR economy OR animals OR insects)
**Web of Science**	TS=("bakery products" OR "bakery product" OR breadmaking OR bread OR bread*)<br>**AND**<br>TS=("gluten free" OR “gluten replacers” OR "without gluten" OR "gluten free flours" OR "gluten free flour")<br>**AND**<br>TS=(food OR "food science" OR nutrition* OR technological OR “experimental studies” OR experimental)<br>**NOT**<br>TS=(cake* OR biscuit* OR brownie* OR danish OR "cinnamon Roll" OR cookie* OR croissant* OR donut OR muffin* OR pasta OR beverage OR beverages OR cracker OR snacks OR extruded OR dessert* OR pizza OR meat OR geography OR history OR philosophy OR anthropology OR economy OR animals OR insects OR “cross-sectional studies” OR “cross-sectional” OR “cohort studies” OR cohort OR “case-control studies” OR “case-control” OR “clinical trial”)
**Wiley online library**	("bakery products" OR "bakery product" OR breadmaking OR bread OR bread*) in All Fields<br>**AND** ("gluten free" OR “gluten replacers” OR "without gluten" OR "gluten free flours" OR "gluten free flour" OR maize OR zein OR rice OR "rice flour" OR "rice starch" OR sorghum OR pseudocereal OR quinoa OR amaranth OR buckwheat OR starch OR corn OR "corn flour" OR "corn starch" OR potato OR "potato starch" OR manioc OR "manioc flour" OR "cassava flour" OR cassava OR "cassava starch" OR tapioca OR yam OR "legume flour" OR "legume flours" OR soya OR soy OR carob OR vinal OR bean OR lentil OR chickpea OR pea OR psyllium OR hydrocolloids OR hydrocolloid OR emulsifier OR "green banana" OR "unripe banana" OR "unripe plantain" OR "green plantain" OR "biomass of green banana" OR "green banana biomass" OR "green banana's biomass" OR "unripe plantain flour" OR "unripe plantain's flour" OR "flour of unripe plantain" OR "unripe plantain biomass" OR "biomass four unripe plantain" OR "unripe plantain's biomass" OR "banana flour" OR pectin OR flaxseed OR "Chia seed" OR linseed OR seed yacon OR millet OR chestnut OR "teff flour" OR teff) in All Fields<br>**AND** (food OR "food science" OR nutrition* OR technological OR “experimental studies” OR experimental) in All Fields<br>**NOT** (cake* OR biscuit* OR brownie* OR danish OR "cinnamon Roll" OR cookie* OR croissant* OR donut OR muffin* OR pasta OR beverage OR beverages OR cracker OR snacks OR extruded OR dessert* OR pizza OR meat OR geography OR history OR philosophy OR anthropology OR economy OR animals OR insects OR “cross-sectional studies” OR “cross-sectional” OR “cohort studies” OR cohort OR “case-control studies” OR “case-control” OR “clinical trial”) in All Fields
